# Supplementary material for: Highly efficient nonlinear optical emission from a subwavelength crystalline silicon cuboid mediated by supercavity mode
Source: Nat Commun. 2022 May 18;13:2749. doi: 10.1038/s41467-022-30503-4 (PMC9117321; doi:10.1038/s41467-022-30503-4)
Supplement: Supplementary file 1 — Supporting information [file 41467_2022_30503_MOESM1_ESM.pdf]

Supplementary Materials

Highly efficient nonlinear optical emission from a subwavelength crystalline silicon cuboid mediated by supercavity mode

Mingcheng Panmai,[1#] Jin Xiang,[1#] Shulei Li,[1] Xiaobing He,[1] Yuhao Ren,[2] Miaoxuan Zeng,[3] Juncong She,[3] Juntao Li,[2*] and Sheng Lan[1*]

[1]*Guangdong Provincial Key Laboratory of Nanophotonic Functional Materials and Devices, School of Information and Optoelectronic Science and Engineering, South China Normal University, Guangzhou 510006, People's Republic of China*

[2]*State Key Laboratory of Optoelectronic Materials and Technologies, School of Physics, Sun Yat-sen University, Guangzhou 510275, People's Republic of China*

[3]*State Key Laboratory of Optoelectronic Materials and Technologies, Guangdong Province Key Laboratory of Display Material and Technology, School of Electronics and Information Technology, Sun Yat-sen University, Guangzhou 510275, People's Republic of China*

**Corresponding author:* lijt3@mail.sysu.edu.cn and slan@scnu.edu.cn

Table of contents

1. Sample fabrication
2. Morphology characterization
3. Evolutions of optical modes with geometrical parameters
4. Influences of SiO_2 layer
    4.1 Influence of SiO_2 layer on quasi-BIC
    4.2 Anisotropic oxidation of Si/SiO_2 cuboid
    4.3 Influence of SiO_2 layer on TPA
5. Methods for determining quasi-BIC
    5.1 Based on quality factor
    5.2 Based on Fano asymmetry parameter *q*
    5.3 Based on phase difference
    5.4 Based on eigenmode
    5.5 Based on scattering intensity
6. Effects of optical doping on quasi-BIC: numerical simulation
7. Effects of optical doping on quasi-BIC: experimental observation
8. Evolutions of optical modes for Si/SiO_2 cuboids with different widths
9. Dependence of luminescence intensity on pulse energy
    9.1 CCD images of luminescence
    9.2 Luminescence spectra below and above the threshold
10. Stability of Si/SiO_2 cuboid after luminescence burst
11. Literature survey for quantum efficiencies of Si-based materials
12. Estimation of quantum efficiency
13. Polarization and radiation pattern of Si/SiO_2 cuboid
14. Luminescence lifetimes of Si/SiO_2 cuboid
15. Size dependence of luminescence intensity
16. Luminescence from arrays of Si/SiO_2 cuboids
17. Optical characterization

Note 1 Sample fabrication

The Si/SiO_2 cuboids studied in this work were fabricated by using a Si-on-sapphire (SOS) wafer obtained by using wafer bonding technique. The SOS wafer was composed of a 230-nm-thick c-Si and a 500-μm-thick sapphire. In Fig. S1, we show the flow chart for the fabrication of Si/SiO_2 cuboids.

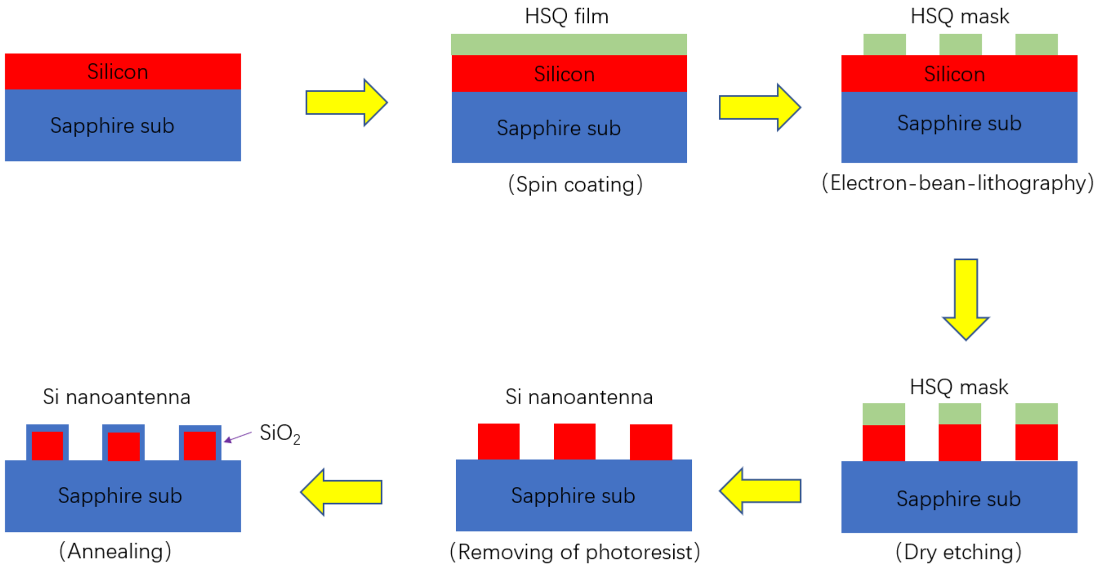

Fig. S1 Flow chart for the fabrication of Si/SiO2 cuboids. Here, HSQ represent hydrogen silsesquioxane, a negative resist.

Note 2 Morphology characterization

In the fabrication process, Si/SiO_2 cuboids with the same geometrical parameters (length and width) were arranged as a 3×3 array, as shown in Fig. S2a, where l and w represent the length and width of the Si/SiO_2 cuboids in the array, respectively. The distance between the two neighboring Si/SiO_2 cuboids was designed to be d = 10 μm so that each Si/SiO_2 cuboid is isolated from its neighbors. The scanning electron microscope (SEM) image of an array of Si/SiO_2 cuboids is shown in Fig. S2b. The SEM image of a typical Si/SiO_2 cuboid with l = 400 nm and w = 190 nm is shown in Fig. S2c. Based on the SEM image, the actual length and width of the Si/SiO_2 cuboid was estimated to be l = 395 nm and w = 192 nm, implying a fabrication tolerance of ~10 nm.

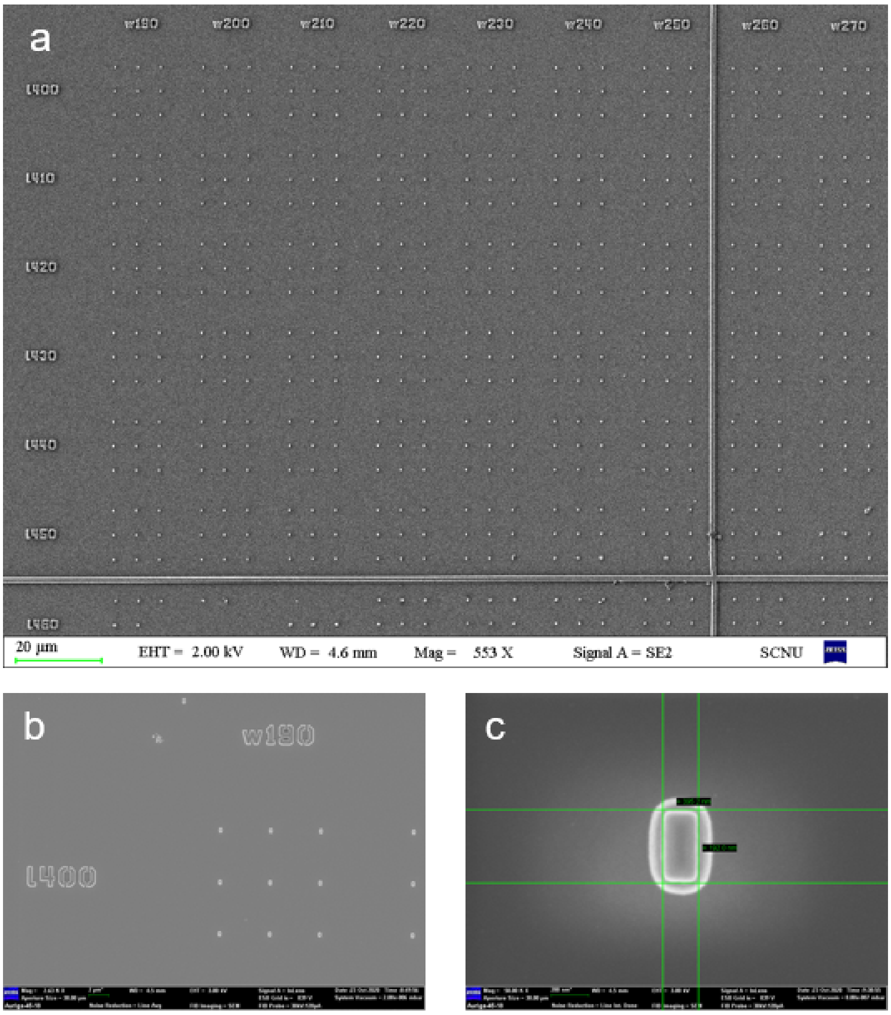

Fig. S2 (a) SEM image of Si/SiO_2 cuboids with different geometrical parameters, which are arranged as 3 × 3 arrays. (b) SEM image of an array of Si/SiO_2 cuboids with l = 400 nm and w = 190 nm. (c) Magnified SEM image of a single Si/SiO_2 cuboid.

Note 4 Evolutions of optical modes with geometrical parameters

In Fig. S3, we examined the evolutions of the optical modes supported by a Si cuboid with w' = 120 nm and h' = 180 nm with increasing length (l') based on the scattering spectra calculated for Si cuboids with different lengths. One can easily identified the ED and MD modes which are nearly independent on the length of the Si cuboid and the F-P mode which is shifted to longer wavelengths with increasing length of the Si cuboid. The refractive index of Si was taken from Aspnes[1].

Here, the near-fields of the ED and MD modes are characterized by using the electric (E_x) and magnetic field (H_z) distributions, as shown in Fig. S3. The near-field of the BIC is characterized by using the magnetic field distribution in the z direction (H_z).

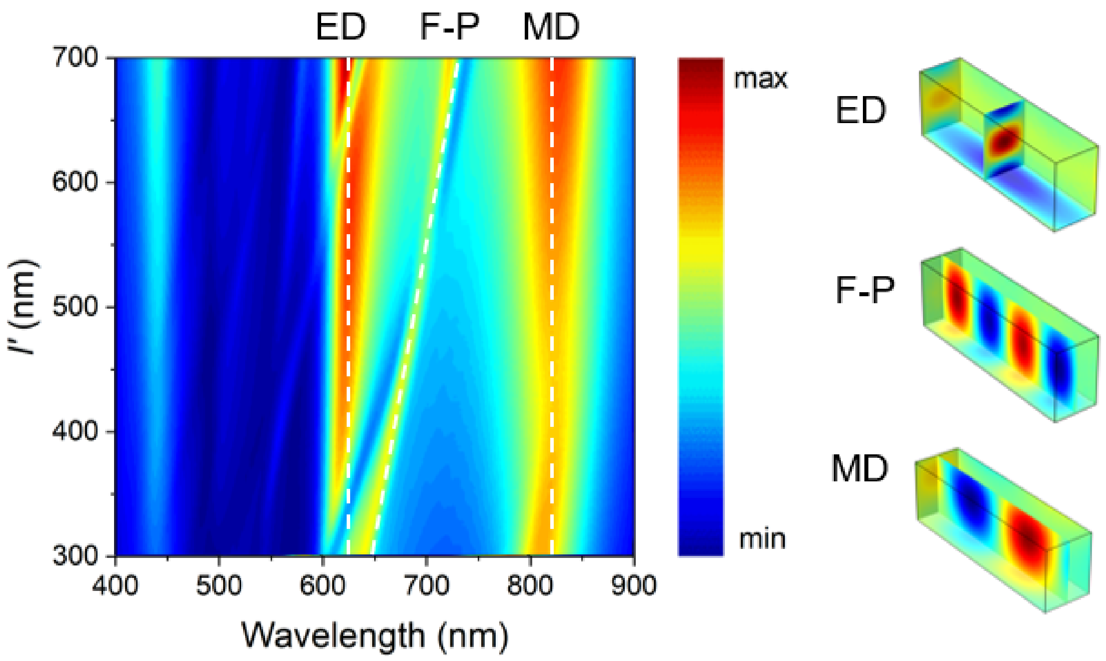

Fig. S3 Evolution of the optical modes (ED, MD, and MO) with increasing length of the Si cuboid. Here, ED, F-P, MD, MO represent electric dipole, Fabry-Perot mode, magnetic dipole, and magnetic octupole respectively.

In Fig. S3, the optical mode supported by the Si cuboid, which depends on the length of the Si cuboid, is indicated by the dashed curve. It is a Fabry-Perot (F-P) mode (MO) whose magnetic field distribution at the quasi-BIC wavelength is shown in the right panel. A standing wave along the y direction is observed, implying that the resonant wavelength of this mode is dependent on the length of the Si cuboid.

Note 4 Influences of SiO_2 layer

4.1 Influence of SiO_2 layer on the quasi-BIC

In order to examine the influences of a SiO_2 layer of on the scattering spectrum and the formation of quasi-BIC, we calculated the scattering spectra for Si/SiO_2 cuboids with different lengths, as shown in Fig. S4a. For comparison, the corresponding spectra for Si cuboids without SiO_2 layers are provide in Fig. S4d. It is found that the evolutions of the optical modes (ED, MD and MO) and the formation of the quasi-BIC are not affected by the introduction of a SiO_2 layer. In Fig. S4b,e, we show the scattering spectra of the Si/SiO_2 and Si cuboids in which the quasi-BIC is clearly resolved. Apart from the slight shift of the resonant wavelength, the quasi-BIC with a similar quality factor as well as a similar magnetic field distribution (see Fig. S4c,f) is obtained in the Si/SiO_2 cuboid. These results indicate clearly that the introduction of a SiO_2 has negligible influences on the formation of quasi-BICs and their optical properties.

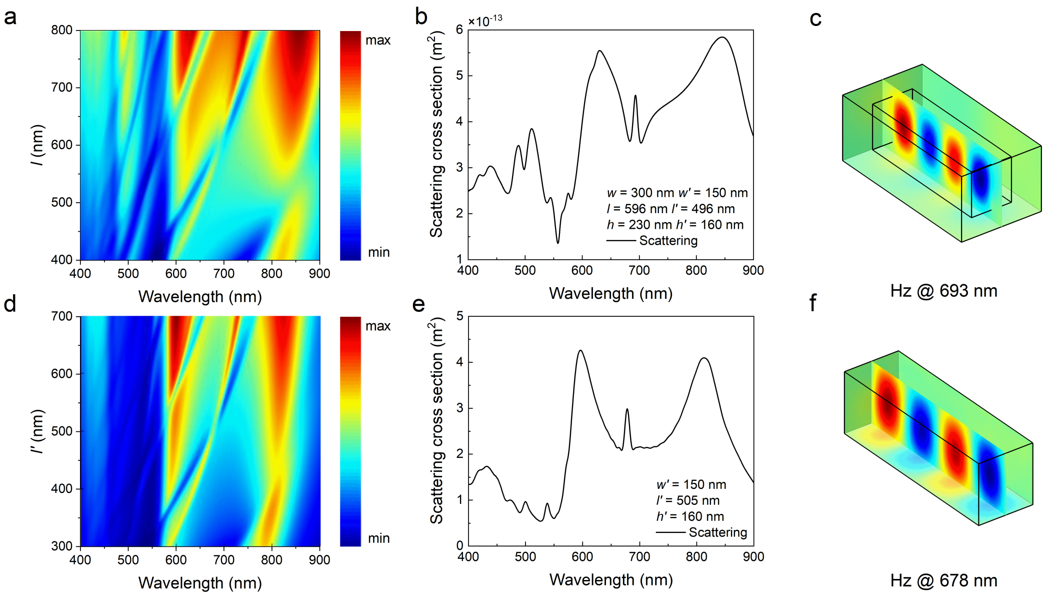

Fig. S4 Evolutions of the optical modes with increasing length (l) of the Si/SiO_2 (a) and Si (d) cuboids. The scattering spectra of the Si/SiO_2 and Si cuboids that support the quasi-BICs are shown in (b) and (e). The magnetic field distributions (H_z) in the Si/SiO_2 and Si cuboids at the quasi -BICs are presented in (c) and (f), respectively.

4.2 Anisotropic oxidation of Si/SiO_2 cuboids

In this work, the dimensions of Si/SiO_2 can be measured with an accuracy of ±10 nm based on the SEM observations. However, the thicknesses of the SiO_2 layers in the three directions cannot be accurately determined. In Fig. 2d, the quasi-BIC is observed at ~696 nm in the scattering spectrum. If we assume that the thicknesses of the SiO_2 layers are the same in all directions (i.e., $t_x = t_y = t_z = t$ and $l = l' + 2t$, $w = w' + 2t$, $h = h' + t$), the quasi -BIC is found at ~625 nm for $t = 85$ nm. In this case, the quasi-BIC observed in the experiment does not match well with that predicted in the numerical simulation. Although the existence of the SiO_2 layer does not influence the formation of the quasi-BIC, it leads to the shift of the resonant wavelength of the quasi-BIC. If we assume an anisotropic oxidation of Si/SiO_2 cuboids in the fabrication of Si/SiO_2 cuboids (i.e., the thicknesses of the SiO_2 layers in the three directions are different), however, the resonant wavelength of the quasi-BIC calculated for the Si/SiO_2 cuboid can be in good agreement with the measured one, as shown in Fig. S5a.

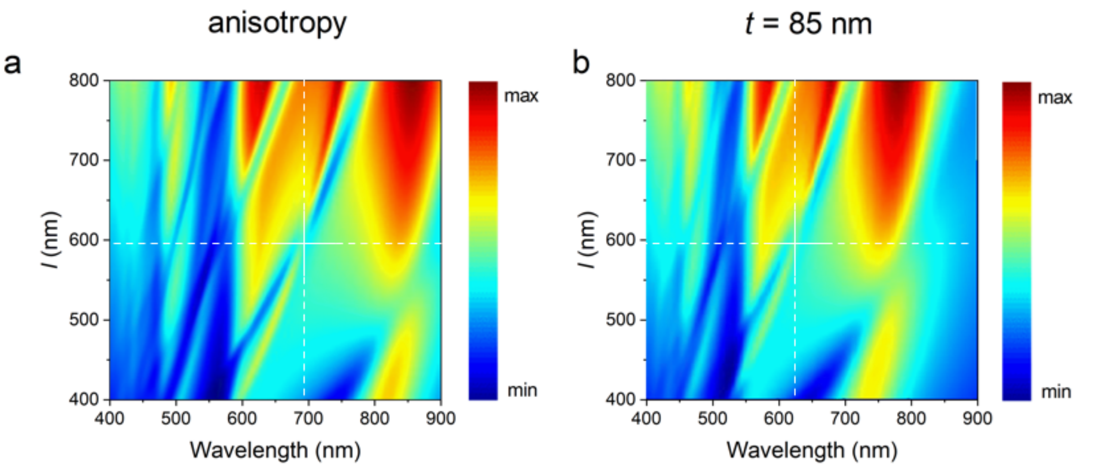

Fig. S5 Evolutions of the optical modes with increasing length of the Si/SiO_2 cuboid. (a) The thicknesses of SiO_2 layers in the x, y, z directions are chosen to be $t_x = 75$ nm, $t_y = 50$ nm, and $t_z = 70$ nm. (b) The thicknesses of thicknesses of SiO_2 layers in the x, y, z directions are chosen to be the same $t_x = t_y = t_z =$ 85 nm.

4.3 Influence of SiO_2 layer on TPA

For a Si/SiO_2 cuboid, the electric field is mainly confined in Si because of the low refractive index (~1.45) of SiO_2. Thus, the influence of the thin SiO_2 on the two-photon-induced absorption (TPA) of the Si/SiO_2 cuboid is almost negligible. In Fig. 3a, we present the TPA spectra calculated for Si/SiO_2 cuboids with different lengths. For comparison, we also calculated the TPA spectra of Si cuboids, as shown in Fig. S6a. It is found that the enhancement factors for TPA are almost the same in the two cases, verifying that the influence of the SiO_2 layer on the TPA is negligible. In Fig. S6b,c, we present the scattering and TPA spectra calculated for a Si cuboid (without SiO_2 layer) with l = 572 nm and w = 180 nm. The measurement results for Si/SiO_2 cuboids with similar sizes of inside Si cuboids are shown in Fig. S6d,e. Except the reduced scattering intensities observed at the long wavelengths (due mainly to the low quantum efficiencies of the detector at long wavelengths), the simulation results are in qualitatively agreement with the experimental observations.

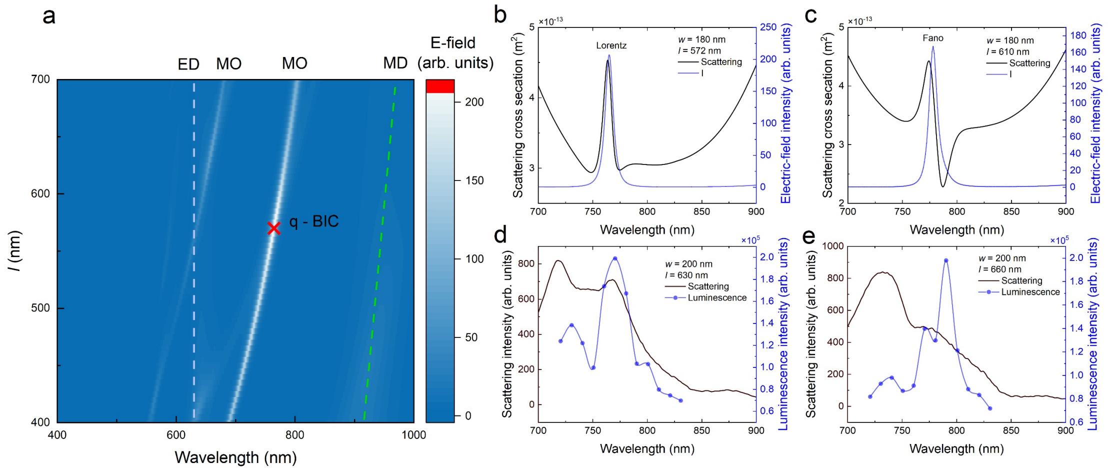

Fig. S6 a, TPA spectra ($\frac{1}{V}\int|E|^4\,\mathrm{d}V$ spectra) calculated for Si cuboids with w' = 180 nm and different lengths. ED, MD, MO represent electric dipole, magnetic dipole, and magnetic octupole respectively. The TPA and scattering spectra calculated for Si cuboids with l' = 572 and 610 nm are shown in **b** and **c**. The excitation spectra measured for Si/SiO_2 cuboids with l = 630 and 660 nm are shown in **d** and **e**, respectively. In each case, the scattering spectrum is also provided for reference.

Note 5 Methods for determining quasi-BIC

5.1 Based on quality factor

The most important feature of a quasi-BIC is the achievement of the largest quality (Q) factor when the structure parameter of the Si/SiO_2 cuboid is changed[2]. In Fig. S7a,b, we show the dependence of the Q factor of a Si/SiO_2 cuboid on its length (l) (with fixed w) or aspect ratio (l/w) obtained by using two simulation methods described in the following. In both cases, it can be seen that the largest Q factor is observed in a Si/SiO_2 cuboid with (l = 596 nm and w = 300 nm). The geometrical parameters of the Si/SiO_2 are exactly the same as those used in Fig. 2b.

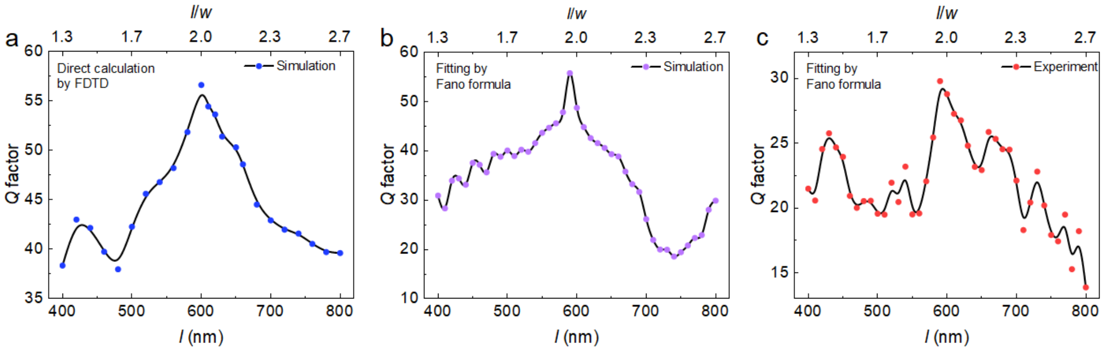

Fig. S7 (a) Dependence of the Q factor on the length or aspect ratio (l/w) of the Si/SiO_2 cuboid calculated by using (a) numerical simulation based on the FDTD method, (b) fitting of the simulated scattering spectra, and (c) fitting of the measured scattering spectra of Si/SiO_2 cuboids.

In order to calculate the eigenmodes and the quality factor of a Si/SiO_2 cuboid, we placed more than 45 point field-time monitors inside the Si/SiO_2 cuboid to obtain the local time evolutions of the electric and magnetic fields. When the Si/SiO_2 cuboid is irradiated by a plane wave (ultrashort pulse), the physical quantities obtained by the field-time monitors include electric field vector, magnetic field vector, Poynting vector, spectrum, oscillation frequency, quality factor, and decay constant etc. In this way, we are able to obtain the quality factors of different modes supported by a Si/SiO_2 cuboid at a specific location from the field-time monitors. When the number of such field-time monitors inside the Si/SiO_2 cuboid is sufficient, we can obtain the eigenmodes of the Si/SiO_2 cuboid and the corresponding decay constants.

When dealing with resonators with high quality factors, we need more data in frequency domain, implying a small time interval. As a result, the mesh size in FDTD simulation is quite small. In the numerical simulations, we set 5001 frequency points in the wavelength range of 400–900 nm and chose a mesh size of 1.0 nm.

In Fig. S7c, we show the dependence of the Q factor on the aspect ratio (*l*/*w*) obtained by fitting the scattering spectra measured for Si/SiO_2 cuboids. In this case, we used Fano formula to fit the scattering spectra and extracted the Q factors by single-peak fitting. We employed the Levenberg-Marquardt algorithm in SciPy software library to perform the calculation with MATLAB.

5.2 Based on Fano asymmetry parameter *q*

Another evidence of the quasi-BIC is based on the evolution of Fano asymmetric parameter *q*. In Fig. S8a, we present the evolution of the scattering spectra measured for Si/SiO_2 cuboids with different lengths ranging from l = 530 nm to l = 650 nm. In each case, the Fano resonance originating from the interference between the ED/MD and MO modes is marked by orange color. With increasing length of the Si/SiO_2 cuboid, it is found that the asymmetric Fano lineshape with a negative *q* evolves into a symmetric Lorentz lineshape at l = 590 nm. After that, a Fano lineshape with a positive *q* is observed. The Q factor of the Fano resonance can be derived by fitting the scattering spectra (orange color) with Fano formula. The dependence of the Q factor on the length or aspect ratio of the Si/SiO_2 cuboid is shown in Fig. S8b. It is noticed that the largest Q factor is achieved at the quasi-BIC of the Si/SiO_2 cuboid with l = 590 nm. In Fig. S8c, we plot the dependence of the *q* value extracted from the fitting of the Fano lineshape on the length or aspect ratio of the Si/SiO_2 cuboid. A transition of the *q* value from positive to negative is observed at l = 590 nm, where a symmetric Lorentz lineshape with $q \to \infty$ is observed.

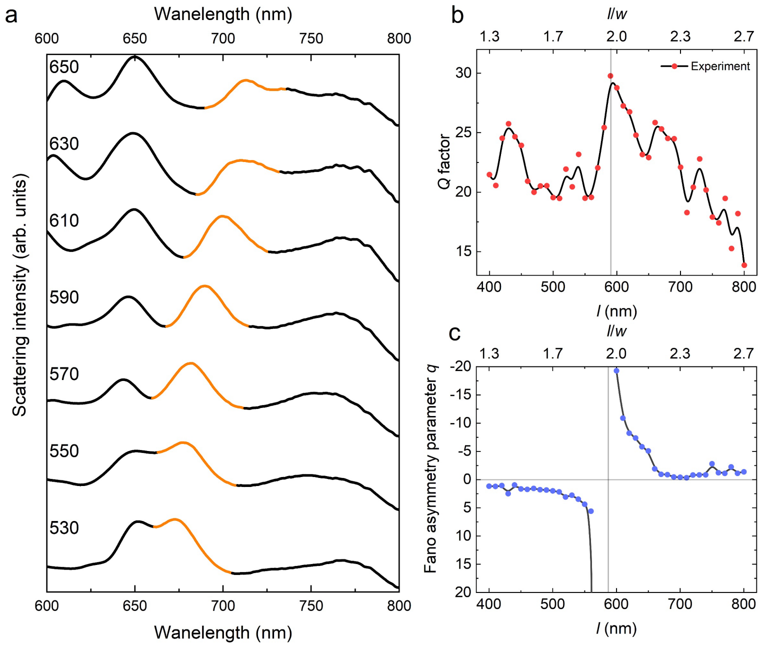

Fig. S8 (a) Scattering spectra measured for Si/SiO_2 cuboids with different lengths. (b) Dependence of the Q factor extracted from the fitting of the Fano resonance on the length or aspect ratio of the Si/SiO_2 cuboid. (c) Dependence of the *q* value from the fitting of the Fano resonance on the length or aspect ratio of the Si/SiO_2 cuboid.

5.3 Based on phase difference

The Fano lineshape originating from the interference of two optical modes depends strongly on the phase difference between them. When the phase difference is equal to 0, π/2 or π, the Fano lineshape will evolve into a symmetric Lorentz lineshape[2]. The criterion for quasi-BIC is fulfilled when the phase difference is equal to 0 or π. In Fig. S9, we show the scattering spectra calculated for Si cuboids with $w' = 120$ nm, $h' = 180$ nm, and $l' = 450$, 525, and 600 nm. In each case, the phase difference between the ED and MO modes is also provided.

For the Si cuboid with $l' = 525$ nm, the peak in the $(\int|E|^4 dV)/V$ spectrum coincides with the scattering peak, which appears as a symmetric Lorentz lineshape. In addition, the phase difference between the ED and MO modes is found to be 0. Therefore, this scattering peak is attributed to the quasi-BIC of the Si cuboid.

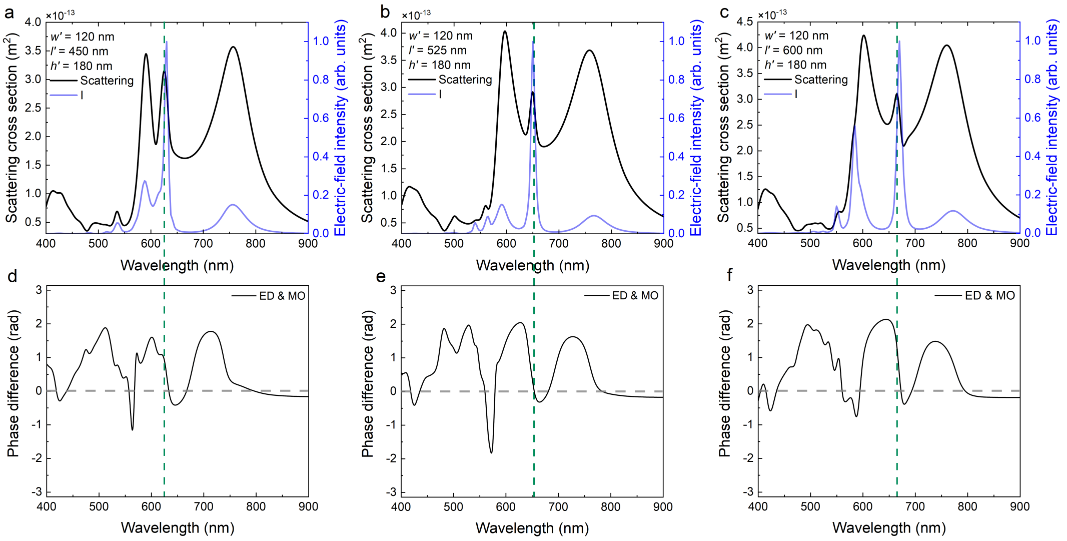

Fig. S9 Scattering spectra calculated for Si cuboids with different lengths of $l = 450$, 525, and 600 nm (upper panel). In each case, the $(\int|E|^4 dV)/V$ spectrum is also provided to characterize the electric field enhancement. Also shown is the phase difference between the ED and MO modes calculated for the corresponding Si cuboid (lower panel). Here, ED, MD, MO represent electric dipole, magnetic dipole, and magnetic octupole respectively.

For the Si cuboid with $l' = 450$ or 600 nm, the scattering peak appears as an asymmetric Fano lineshape. The phase difference between the ED and MO modes is not equal to 0. Moreover, it is noticed that there is a wavelength shift between the peak of the $(\int|E|^4 dV)/V$ spectrum and that of the scattering spectrum.

5.4 Based on eigenmode

One of the criterions for judging the BIC is the disappearance of the corresponding Fourier coefficient[3]. Here, we placed randomly distributed electric dipole sources in a Si cuboid to excite its eigenmodes. Meanwhile, a time monitor is used to record the time evolution of the local electric field inside the Si cuboid. The Fourier transformation of the electric field in time domain gives rise to the eigenmodes of the Si cuboid in frequency domain. In Fig. S10a, we present the scattering spectra calculated for Si cuboids with different length l' (w'= 120 nm, h' = 180 nm). The electric and magnetic field intensities extracted from the time monitor are shown in Fig. S10b,c. The quasi-BIC is revealed at the wavelength (or frequency) where the electric mode disappears and the magnetic mode reaches its maximum value.

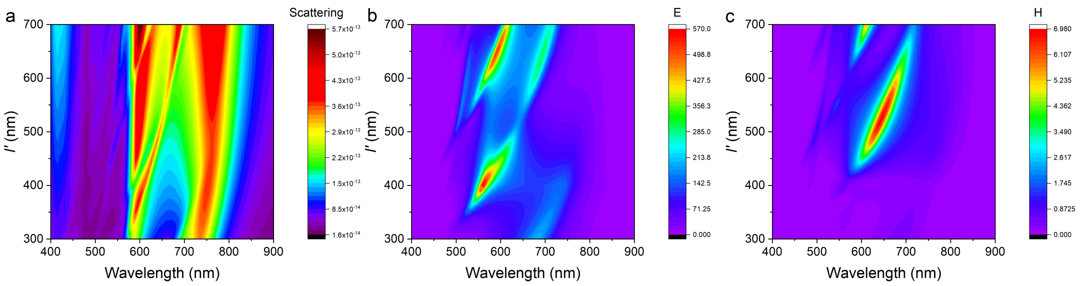

Fig. S10 (a) Scattering spectra calculated for Si cuboids with different lengths (w' = 120 nm, h' = 180 nm). The electric and magnetic field intensities extracted from the time monitor are shown in (b) and (c), respectively.

5.5 Based on scattering intensity

We examined the evolution of the scattering spectrum of the Si cuboid with increasing length and extracted the dependence of the intensity of the Fano peak on the length of the Si cuboid, as shown in Fig. S11. The minimum intensity of the Fano peak is observed for the Si cuboid with $l' = 510$ nm, $w' = 150$ nm, and $h' = 160$ nm. It implies that a quasi-BIC is achieved in the Si cuboid with $l' = 510$ nm. In this case, the radiation of the Si cuboid comes only from the magnetic mode and those originating from the electric modes are suppressed.

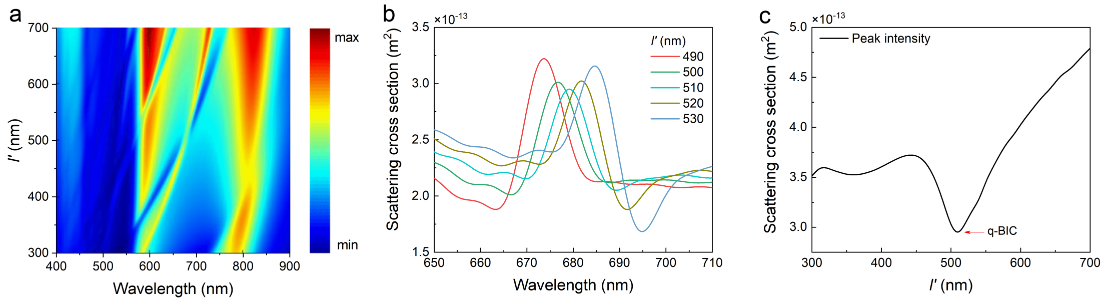

Fig. S11 (a) Scattering spectra calculated for Si cuboids with different lengths (w' = 150 nm, h' = 160 nm). (b) Scattering spectra calculated for Si cuboids with l' = 490, 500, 510, 520, and 530 nm. (c) Dependence of the scattering intensity of the Fano peak on the length of the Si cuboid.

Note 6 Effects of optical doping on quasi-BIC: numerical simulation

In Fig. S12, we present the scattering spectra calculated for a Si cuboid with l' = 560 nm with different densities of injected carriers. In each case, the $(\int|E|^4 dV)/V$ spectrum for the Si cuboid is also provided. It is remarkable that the quasi-BIC collapse rapidly when the density of injected carriers exceeds 10^{20} cm^{-3}. Accordingly, the two-photon-induced absorption, which is characterized by $(\int|E|^4 dV)/V$, also drops dramatically by nearly two orders of magnitude when the carrier density is increased to 10^{19} cm^{-3}. This behavior implies that the quasi-BI in the Si cuboid can only be employed to inject high-density carriers. It is not suitable for being used as the cavity mode for lasing.

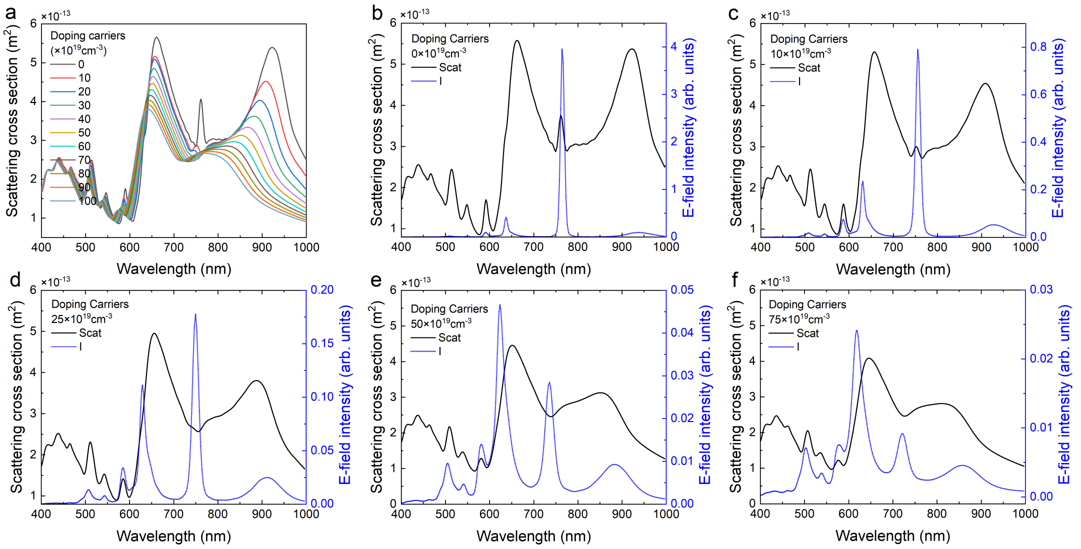

Fig. S12 Evolution of the scattering spectrum of a Si cuboid with l' = 560 nm, w' = 180 nm, and h' = 180 nm with increasing density of the injected carriers. The corresponding evolution of the $(\int|E|^4 dV)/V$ spectrum is also provided.

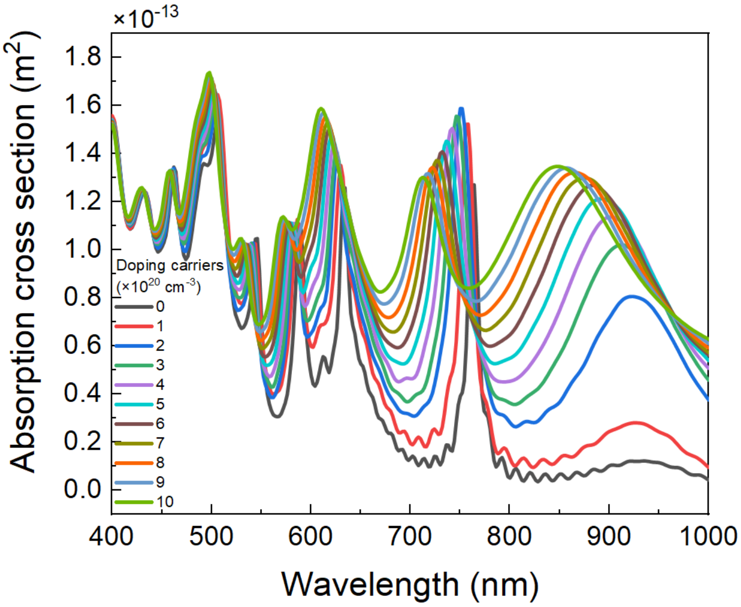

Fig. S13 Evolution of the absorption spectrum with increasing carrier density calculated for a Si cuboid with l' = 560 nm, w' = 180 nm, and h' = 180 nm.

In Fig. S13, we present the absorption spectra calculated for a Si/SiO_2 cuboid (l' = 560 nm and w' = 180 nm) at different injected carrier intensities. In this case, one can identify four absorption peaks in the absorption spectrum. With increasing carrier intensity, a blueshift is observed for all absorption peaks. For the absorption peak corresponding to the quasi-BIC (λ ~760 nm), the absorption increases initially with increasing carrier density and reaches a maximum rapidly at 2.0 x 10^{20} cm^{-3}. Then, it begins to decrease with increasing carrier density. The reduction of the absorption is caused by the quenching of the quasi-BIC at high doping levels. In contrast, a rapid increase in absorption is observed for the other three peaks and the saturation of absorption is observed at high doping levels.

Note 7 Effects of optical doping on BIC: experimental observation

In Fig. S14a,c, we present the scattering spectra measured for Si/SiO_2 cuboids with different lengths. The corresponding luminescence spectra for the Si/SiO_2 cuboids are shown in Fig. S14b,d. The excitation wavelength was chosen to be 785 nm, which is close to the magnetic dipoles of the Si/SiO_2 cuboids. Thus, carriers were injected into the Si/SiO_2 cuboids through the magnetic dipoles. In each figure, the evolution of the F-P mode is indicated by a dashed line. For Si/SiO_2 cuboids with l < 500 nm, one can identify the F-P modes in the luminescence spectra as the enhancements of the luminescence. However, it is noticed that the enhancement in the luminescence disappears in the Si/SiO_2 cuboid supporting the quasi-BIC (l ~ 590 nm) because of the collapse of the quasi-BIC induced by optical doping. The F-P mode is revealed in the luminescence spectra of the Si/SiO_2 cuboids with l > 610 nm.

This behavior implies that the quasi-BIC will be destroyed by injecting high-density carriers into the Si/SiO_2 cuboid, which modified significantly the imaginary part of the complex refractive index of Si. Therefore, the quasi-BIC cannot be used as the cavity mode for lasing. However, it can be employed to inject high-density carriers into the Si/SiO_2 cuboid, similar to the magnetic dipole.

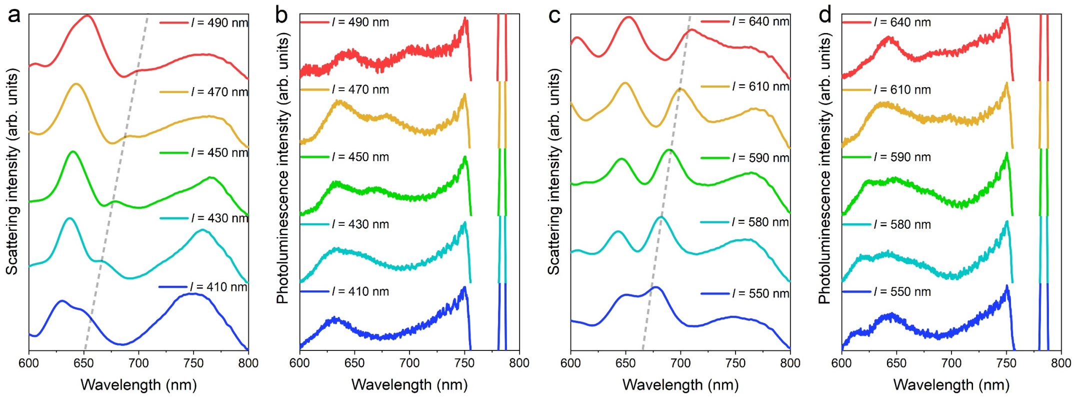

Fig. S14 Scattering (a,c) and luminescence (b,d) spectra measured for Si/SiO_2 cuboids with different lengths ranging from 410 to 640 nm.

Note 8 Evolutions of optical modes for Si/SiO_2 cuboids with different widths

In Fig. S24, we show the scattering spectra measured for Si/SiO_2 cuboids with different lengths. In this case, the widths of Si/SiO_2 cuboids are fixed at w = 340 nm and the quasi-BIC appears at ~720. The luminescence properties of the Si/SiO_2 cuboid at the quasi-BICs were examined, as shown in Fig. 5 in the main text and Fig. S6 in the Supplementary Note 4.3. It is noticed that the MD modes appear in the wavelength range of 750–800 nm for Si/SiO_2 cuboids with $l < 450$ nm. The MD mode is red shifted with increasing length of the Si/SiO_2 cuboid. For Si/SiO_2 cuboids with $l > 450$ nm, the MD modes are invisible because of the small power of the lamp and the low quantum efficiency of the detector. The evolution of the scattering spectrum with increasing length of the Si/SiO_2 cuboid with w = 370 nm is presented in Fig. S15b. The excitation spectra of the Si/SiO_2 cuboids around the quasi-BIC are presented in Fig. S6 (see Supplementary Note 4.3).

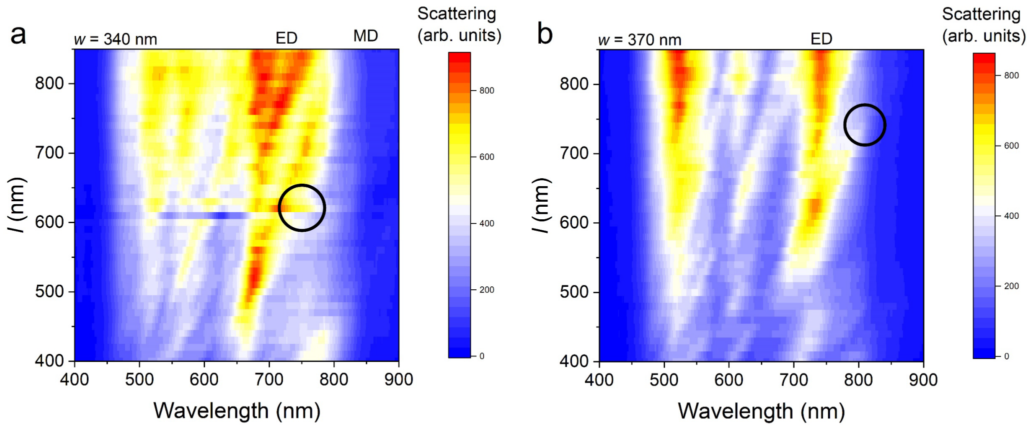

Fig. S15 Scattering spectra measured for Si/SiO_2 cuboids with different lengths. (a) w = 340 nm. (b) w = 370 nm. Here, ED and MD represent electric dipole and magnetic dipole.

Note 9 Excitation pulse energy dependence of luminescence intensity

9.1 CCD images of luminescence

In Fig. S16, we show the luminescence images of a Si/SiO_2 cuboid with a larger volume (l = 620 nm, w = 340 nm, and h = 230 nm), which was excited by using femtosecond laser light at different pulse energies, under a dark- and bright-field microscope. They were recorded by using a coupled charge device (CCD) attached on the microscope. The excitation wavelength was chosen to be 720 nm. It can be seen that the brightness of the Si/SiO_2 cuboid at excitation pulse energy of 6.15 pJ is comparable to that of the illumination light on the background. At excitation pulse energy of ~8.2 pJ, a luminescence cross section as large as ~20 × 20 μm^2 was observed. In this case, the threshold energy (~8.2 pJ) is much larger than that observed in a small Si/SiO_2 cuboid (see Fig. 4 in the main text).

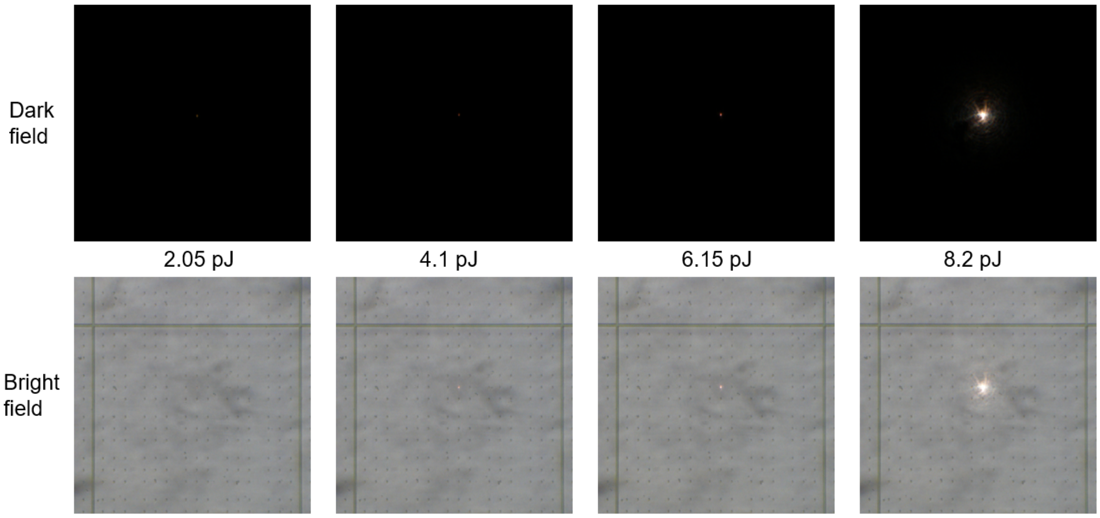

Fig. S16 CCD images of the luminescence from a Si/SiO_2 cuboid excited by using femtosecond laser light with different pulse energies.

9.2 **Luminescence spectra below and above the threshold**

As described in the main text, a dramatic increase in the luminescence intensity is observed when the pulse energy exceeds a threshold. For this reason, the spectral profile below the threshold was not clearly resolved in Fig. 4e. In Fig. S17, we present the luminescence spectra measured for a Si/SiO_2 cuboid with $l = 440$ nm and $w = 260$ nm below and above the threshold. Several peaks corresponding to the enhanced luminescence at the Mie resonances of the Si/SiO_2 cuboid (e.g., ED/EQ/MQ) are observed in both cases.

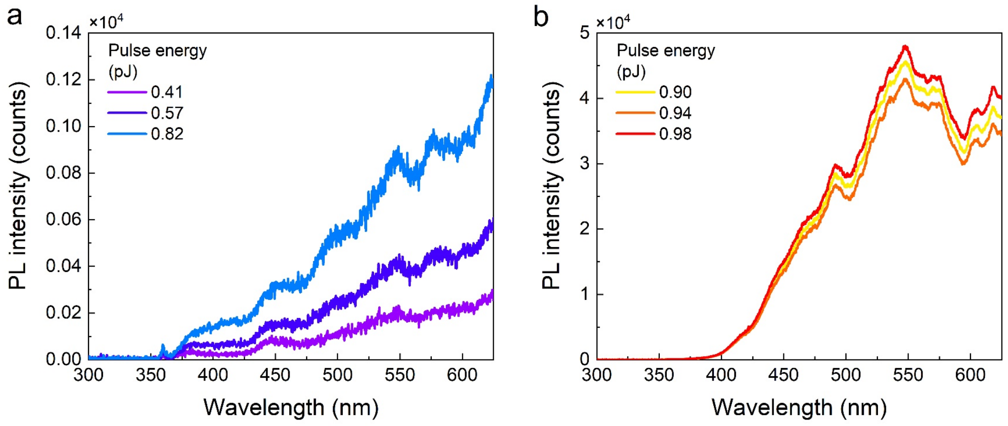

Fig. S17 Luminescence spectra measured for a Si/SiO_2 cuboid with $l = 440$ nm and $w = 260$ nm before (a) and after (b) the threshold pules energy which is 0.90 pJ.

Note 10 Stability of Si/SiO_2 cuboids after luminescence burst

As discussed in the main text, the luminescence burst observed in Si/SiO_2 cuboids originates from the interband radiative recombination of hot electrons. The Auger effect and intrinsic excitation of carriers play important roles in the generation of highly efficient nonlinear optical emission. A large temperature rise, which arises from the thermalization of hot electrons, is expected in Si/SiO_2 cuboids. In order to find out whether the crystalline structure of Si is damaged by the high temperature, we examined the scattering spectra of Si/SiO_2 cuboids before and after the luminescence burst. A typical example is shown in Fig. S18. It was found that the scattering spectrum of the Si/SiO_2 cuboid remains unchanged after the luminescence burst, implying the crystalline structure is not damaged.

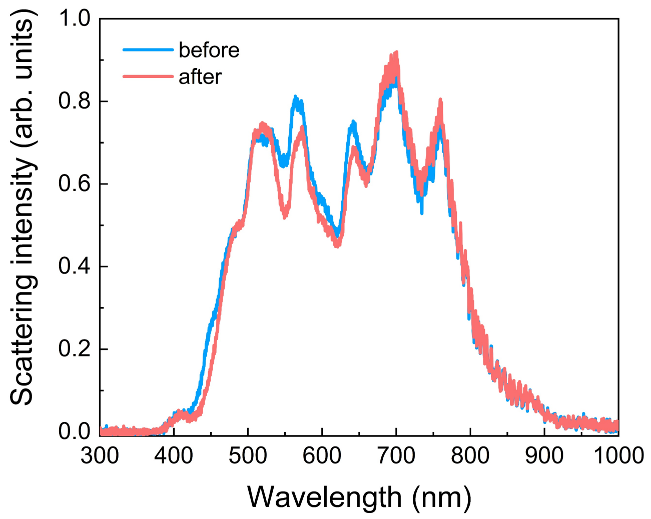

Fig. S18 Scattering spectra measured for a Si/SiO_2 cuboid with l = 600 nm and w = 280 nm before and after the luminescence burst.

Note 11 Literature survey for the quantum efficiencies of Si-based materials

Basically, the quantum efficiency for the photoluminescence of a material η is generally defined as $\eta = N_{pl}/N_{abs}$, where N_{pl} and N_{abs} represent the number of emitted photons and the number of absorbed photons, respectively. This quantity is usually referred to as internal quantum efficiency or quantum yield. A popular expression for quantum yield is $\eta = 1(1+\tau_r/\tau_{nr})$, where τ_r and τ_{nr} denote the radiative and nonradiative recombination times of the material. Therefore, the quantum yield of the material can be derived based on the measurements of τ_r and τ_{nr}. Apart from the internal quantum efficiency, the external quantum efficiency, which is defined as the ratio of the number of photons emitted from a material to the number of exciting photons absorbed by the material, is also used to characterize the photoluminescence from a material. We have made a detailed survey for the quantum efficiencies of Si-based nanomaterials, including Si nanocrystals, quantum dots and porous Si. The material types, preparation methods, quantum efficiency values, and measurements are summarized in Table T1.

In our case, we employed the original definition (i.e., $\eta = N_{pl}/N_{abs}$) to derive the quantum efficiency of Si/SiO_2 cuboids. Since the absorption of Si/SiO_2 cuboids is dominated by nonlinear optical absorption (2PA and 3PA), it makes it possible to extract the number of photons absorbed by a Si/SiO_2 cuboid, described in detail in Supplementary Note 12. In addition, we also considered the directivity of the luminescence and the collection efficiency of the objective when estimating the number of emitted photons. As compared with the methods based on the measurements of τr and τnr, this method gives a more accurate value for the quantum efficiency of Si/SiO_2 cuboids.

Table T1: Internal and external quantum efficiency of silicon-based materials

Materials	Preparation method	Quantum efficiency	Measurement method	Reference
Porous Si (for most samples)	Anodization and stain etching	1%-10%	External quantum efficiency	Ref. (4) (REVIEW)
Single porous Si nanoparticles	Anodization and stain etching	88% (for single) 2.8% (for total)	External quantum efficiency	Ref. (5)
Amorphous Si quantum dots	plasma-enhanced chemical vapor deposition (PECVD)	2×10^{-3} %	External quantum efficiency	Ref. (6)
Amorphous Si nanoparticles	nonthermal low-pressure plasma reactor	1, 2%<br>2, 45%	1, External quantum efficiency<br>2, Internal quantum efficiency	Ref. (7)
Crystalline Si nanoparticles	nonthermal low-pressure plasma reactor	> 40%	External quantum efficiency	Ref. (7)
Si quantum dots	wet-chemical method	4%	External quantum efficiency	Ref. (8)
Si quantum Dots	wet-chemical method	3.7%-11%	External quantum efficiency	Ref. (9)
Si nanocrystals separated by SiO_2 barriers	Plasma-enhanced chemical vapor deposition (PECVD)	10%-19%	External quantum efficiency	Ref. (10)
Silicon Nanocrystals	nonthermal low-pressure plasma reaction	25%-50%	External quantum efficiency	Ref. (11)
Si nanocrystals	nonthermal low-pressure plasma reaction	< 62%	External quantum efficiency	Ref. (12)
Si nanocrystals	non-thermal plasma reaction	60%-70%	External quantum efficiency	Ref. (13)
Si nanocrystals embedded in a SiO_2	low-temperature annealing in ambients of O_2 and H_2	1, 9.3%-27%<br>2, 30%-85%	1, External quantum efficiency<br>2, Internal quantum efficiency	Ref. (14)
Si nanocrystals	annealing dried commercial hydrogen silsesquioxane solution at 1100°C in a slightly reducing atmosphere with 5% H_2.	1, 30%-70%<br>2, 100%	1, External quantum efficiency<br>2, Internal quantum efficiency	Ref. (15)
Si nanocrystals	radio-frequency cosputtering followed by hightemperature annealing at 1100 °C	1, 20%<br>2, 39%	1, External quantum efficiency<br>2, Internal quantum efficiency	Ref. (16)

Si nanocrystals	limited surface oxidation	1, 5%-43%<br>2, 6%-40%	1, External quantum efficiency<br>2, Internal quantum efficiency	Ref. (17)
Si nanocrystals	radiofrequency co-sputtering method	0.5%-20%<br>30%-90%<br>"Step-like"	External quantum efficiency	Ref. (18)
Si nanocrystals	plasma-synthesized	12%-45%	Internal quantum efficiency<br>(Electronic density of states (DOS) calculation by *ab initio* density-functional theory (DFT))	Ref. (19)
Si nanocrystals	wet thermal oxidation	59% ±9%	Internal quantum efficiency<br>(Using LDOS correction)	Ref. (20)
Si nanocrystals	co-sputtering method	40%-100%	Internal quantum efficiency<br>(Using LDOS correction)	Ref. (21)
Si quantum dots (Couple to surface plasmons at the Ag/SiO_2 interface)	annealed for 10 min at 1100 °C	77±17 %	Internal quantum efficiency<br>(Using LDOS correction)	Ref. (22)
Si Nanoparticles	high-pressure microdischarges	30%	External quantum efficiency	Ref. (23)

Note 12 Estimation of quantum efficiency

Basically, the external quantum efficiency for the luminescence of a Si/SiO_2 cuboid can be expressed as follows:

$$EQE = N_1^{pl}/N_1^{ex}, \quad (1)$$

where N_1^{pl} and N_1^{abs} represent the total number of photons emitted from and absorbed by the Si/SiO_2 cuboid, respectively.

In Fig. S19 a,b, we show the optical paths of the excitation laser light (λ ~ 720 nm) and the generated hot electric luminescence in the experimental setup used to simultaneously excite Si/SiO_2 cuboid and to collect/detect the optical signals. In Fig. S19a, it can be seen that the femtosecond laser light is reflected by a dichroic mirror and focused on the Si/SiO_2 cuboid (on a sapphire substrate) by the objective lens of the microscope. The reflected laser light from the Si/SiO_2 cuboid is attenuated by the combination of the dichroic mirror and a long-pass filter with an optical density of OD ~ 2.3×10^{-6}. If we assume that the attenuation coefficient of the whole optical system (except the dichroic mirror + filter) is α, and the gain and quantum efficiency of the charge coupled device (CCD) are g_{ex} and η_{ex}, then the total number of photons detected by the CCD, i.e., N_2^{ex}, can be expressed as follows:

$$N_1^{ex} * r * OD * \alpha * g_{ex} * \eta_{ex} = N_2^{ex}, \quad (2)$$

where N_1^{ex} represents the total number of photons irradiating on the Si/SiO_2 cuboid and r denotes the corresponding reflectivity.

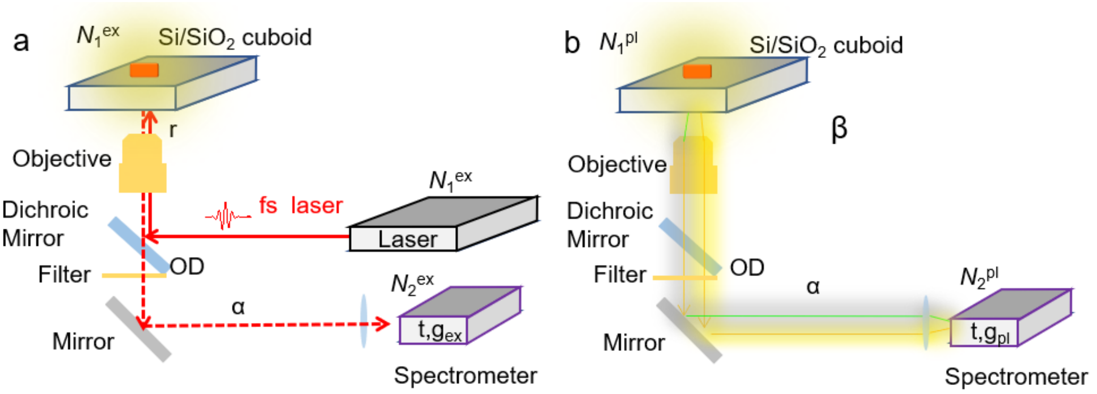

Fig. S19 Schematics showing the optical paths of the excitation laser light (a) and the luminescence emitted from a Si/SiO_2 cuboid (b) in the experiment setup used to estimate the external quantum efficiency of the Si/SiO_2 cuboid.

If we compared the intensity of the reflected light from the substrate without and with the Si/SiO_2 cuboid, a small reduction is observed, as shown in Fig. S21a. This reduction is induced by the linear absorption and scattering of the excitation laser light by the Si/SiO_2 cuboid. When we examined the dependence of the reflected light intensity on the excitation pulse energy (or excitation power), a linear relationship is observed at low excitation pulse energies, as shown in Fig. S22 (see also Fig. 4f in the main text). When the excitation pulse energy exceeds a critical value (or a threshold), a deviation from the linear relationship occurs, implying that the nonlinear absorption (e.g., two-photon-induced absorption or TPA) of the Si/SiO_2 cuboid becomes effective. Apparently, the photons absorbed by the Si/SiO_2 cuboid via TPA can be deduced from the reduction in the reflected light intensity (or the deviation of the reflected light intensity from the linear relationship), as indicated by the arrows in Fig. S22 (see also Fig. 4f).

Therefore, the number of photons absorbed by the Si/SiO_2 cuboid (N_1^{abs}) due to nonlinear processes can be deduced by the number of reduced photons observed in the reflected light (N_2^{abs}) by the following relationship:

$$N_2^{abs} = N_1^{abs} * r * OD * \alpha * g_{ex} * \eta_{ex}. \quad (3)$$

Referring to Fig. S19b, we can obtain the similar relationship between the number of detected photons (N_2^{pl}) in the luminescence and the number of emitted photons from the Si/SiO_2 cuboid (N_1^{pl}), which is given in the following:

$$N_2^{pl} = N_1^{pl} * \beta * \alpha * g_{pl} * \eta_{pl}. \quad (4)$$

Here, the collection efficiency of the objective lens (β) is taken into account. The value depends strongly on the substrate used to support the Si/SiO_2 cuboid. For Si/SiO_2 cuboid located on the sapphire substrate, the average collection efficiency in the visible to near infrared spectral range was found to be $\beta \sim 0.58$, as show in Fig. S20.

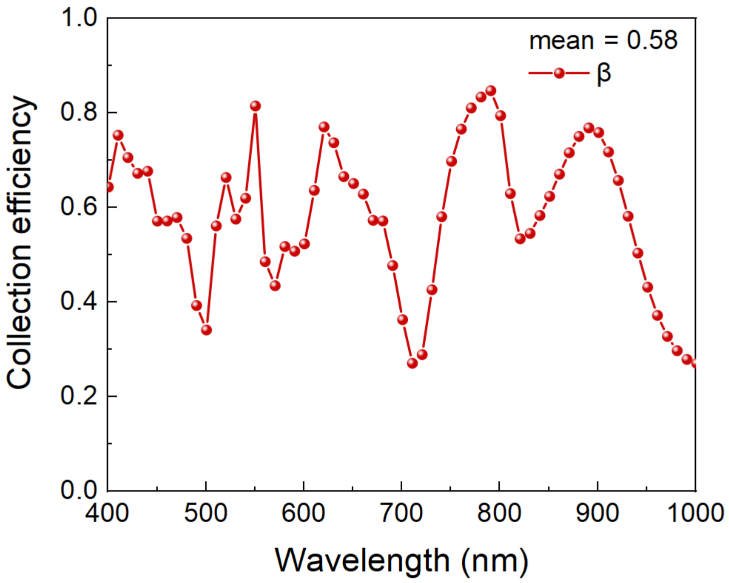

Fig. S20 Collection efficiencies (β) calculated for a Si/SiO_2 cuboid. (l = 440 nm, l' = 340 nm, w = 260 nm, w' = 160 nm, h = 230 nm, h' = 180 nm) at different emission wavelengths.

With the information on the number of photons absorbed by the Si/SiO_2 cuboid (N_1^{abs}) and that emitted from the Si/SiO_2 cuboid (N_1^{pl}), one can readily derive the external quantum efficiency of the hot electron luminescence, which is expressed as follows:

$$EQE = N_1^{pl}/N_1^{abs} = N_2^{pl} * r * OD * \eta_{ex}/(N_2^{abs} * \beta * \eta_{pl}). \quad (5)$$

The external quantum efficiency would be 2*EQE* or 3*EQE* if a two- or three-photon-induced absorption is involved in the generation of carriers. From Eq. (5), it is remarkable that the external quantum efficiency *EQE* is independent on the attenuation coefficient of the optical system (α) because both the reflected laser light and the generated luminescence suffer the same attenuation before reaching the detector. Hence, the attenuation coefficient is cancelled out in the final expression of the external quantum efficiency. However, the information of the attenuation coefficient is necessary if we want to derive the total number of photons absorbed by the Si/SiO_2 cuboid or the total number of photons emitted from the Si/SiO_2 cuboid (see Eq. (3) and Eq. (4)).

Basically, the value of α is determined mainly by the optical coupling between the microscope and the spectrometer, especially the slit width of the spectrometer. In this work, the

slit width was intentionally reduced in order to avoid the saturation of the CCD, which might occur in the burst of luminescence.

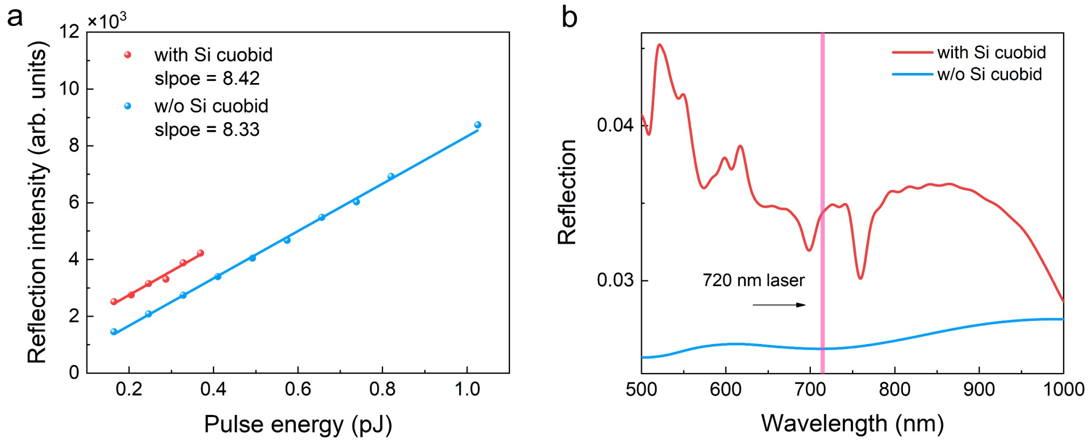

Fig.S21 (a) Dependence of the intensity of the reflected laser light on the pulse energy measured for a sapphire substrate without and with a Si cuboid (l = 440 nm, w = 260 nm). (b) Reflection spectra simulated for a sapphire substrate (d = 500000 nm) without and with a Si/SiO_2 cuboid (l = 440 nm, l' = 340 nm, w = 260 nm, w' = 160 nm, h = 230 nm, h' = 180 nm).

It should be emphasized that we measure the reflection signal of a laser light, which is much stronger than any luminescence, by using a highly sensitive EMCCD. A band-stop filter with a large attenuation coefficient (~10^{-6}) must be used to attenuate the reflected light in order to avoid the damage of the EMCCD. Therefore, the dependence of the reflected light intensity on the excitation light intensity could be accurately determined even at low pulse energies. In Fig. S21a, we present the dependence of the reflected laser light on the pulse energy measured in the cases with and without a Si/SiO_2 cuboid (l = 440 nm, w = 260 nm). In both cases, a linear relationship is observed. It is noticed that the slopes of the two linear relationships are the same and the reflectivity is slightly larger in the presence of the Si/SiO_2 cuboid. The linear relationship remains even at large pulse energies in the absence of the Si/SiO_2 cuboid. Therefore, the slope in the presence of the Si/SiO_2 cuboid obtained at low pulse energies can be calibrated by using that obtained in the absence of the Si/SiO_2 cuboid. We also performed numerical simulations for the reflection from a sapphire substrate with and without a Si/SiO_2 cuboid (l = 440 nm, l' = 340 nm, w = 260 nm, w' = 160 nm, h = 230 nm, h' = 180 nm), as shown in Fig.

S21b. It is remarkable that the simulation results are in good agreement with the experimental observations, implying the feasibility of using this method in measuring the external quantum efficiency of Si/SiO_2 cuboids.

In experiments, we measured the intensity of the excitation laser light reflected from the sample as a function of the excitation pulse, as shown in Fig. S22. A linear relationship between the intensity of the reflected laser light and the excitation pulse energy was observed at low pulse energies. The nonlinear optical absorption can be derived from the deviation of the intensity of the reflected laser light from the linear relationship at high pulse energies, as indicated in Fig. S22. The external quantum efficiency of the luminescence can be estimated by using the formula given in Eq. (5). In our case, the parameters used to derive the external quantum efficiency were $r = 0.15$, OD = 2.3×10^{-6}, $\beta = 0.58$, $\eta_{ex} = 0.8$, $\eta_{pl} = 0.55$. The external quantum efficiency was estimated to be ~13 % at pulse energy of 1.15 pJ where the luminescence burst was observed.

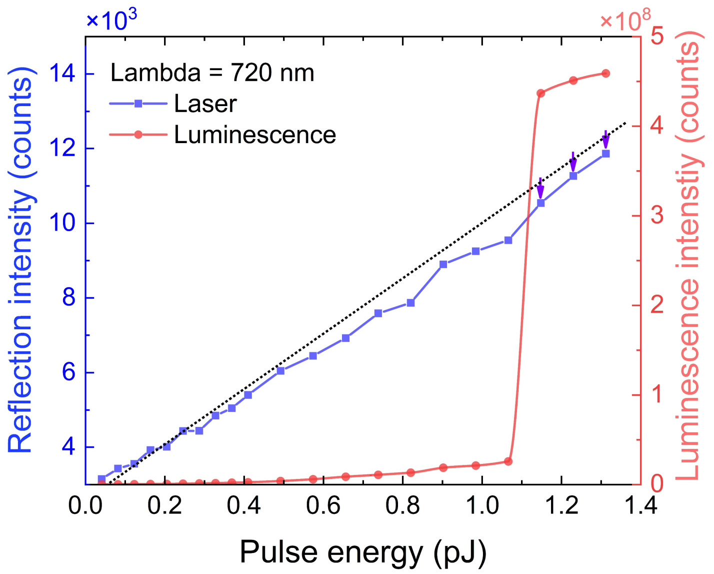

Fig. S22 Dependence of the intensity of the reflected laser light at 720 nm on the excitation pulse energy measured for a Si/SiO_2 cuboid (l = 440 nm, w = 260 nm, h = 230 nm). Also shown is the dependence of the luminescence intensity on the excitation pulse energy from which the luminescence burst was observed at 2.04 pJ. The nonlinear optical absorptions at different excitation pulse energies are indicated by arrows.

Note 13 Polarization and radiation pattern of Si/SiO_2 cuboids

We examined the polarization of the luminescence emitted from Si/SiO_2 cuboids by inserting a polarization analyzer in the collection channel. A typical example is shown in Fig. S23a. In Fig. S23b, we shown the dependence of the luminescence intensity on the polarization angle observed at $\lambda = 600$ nm. It is found that the luminescence of the Si/SiO_2 cuboid exhibits a linear polarization perpendicular to the length of the Si/SiO_2 cuboid. We also simulated the radiation pattern of a Si/SiO_2 cuboid ($l = 720$ nm, $l' = 530$ nm, $w = 360$ nm, $w' = 180$ nm, $h = 230$ nm, $h' = 180$ nm) at $\lambda = 688$ nm, as shown in Fig. S23c. The two-dimensional radiation patterns in the upper and lower backward focal planes. It is found that the radiation of the Si/SiO_2 cuboid is governed by the radiations from ED and MQ, as shown in Fig. 2b in the main text.

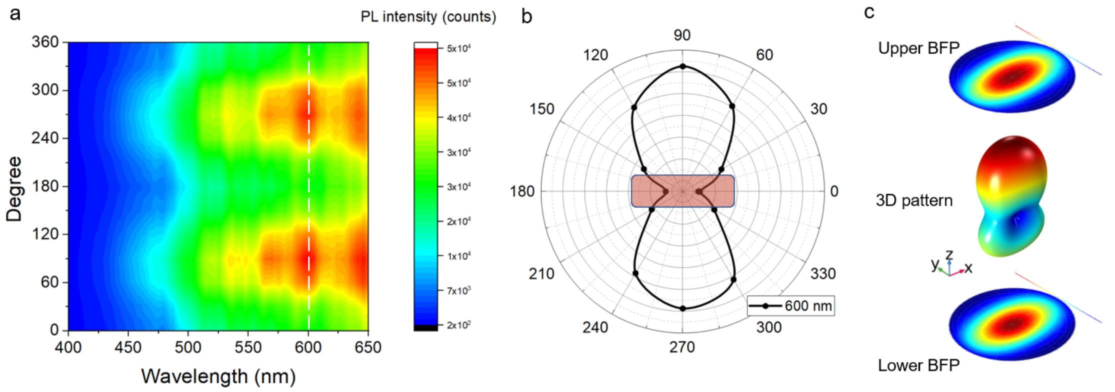

Fig. S23 (a) Luminescence spectra of a Si/SiO_2 cuboid ($l = 720$ nm, $w = 360$ nm) obtained by using a polarization analyzer with different polarization angles. (b) Dependence of the luminescence intensity on the polarization angle observed at $\lambda = 600$ nm. (c) Three-dimensional radiation pattern simulated for a Si/SiO_2 cuboid ($l = 720$ nm, $l' = 530$ nm, $w = 360$ nm, $w' = 180$ nm, $h = 230$ nm, $h' = 180$ nm) at $\lambda = 688$ nm. Also shown are the two-dimensional radiation patterns in the upper and lower back focal planes, BFP.

Note 14 Luminescence lifetimes of Si/SiO_2 cuboids

We have performed luminescence decay measurement for Si/SiO_2 cuboids. A typical example obtained for a Si/SiO_2 cuboid (l = 600 nm, w = 340 nm) is shown in Fig. S24. The luminescence lifetimes extracted by fitting the luminescence are found to be ~110 ps and ~49 ps before and after the luminescence burst.

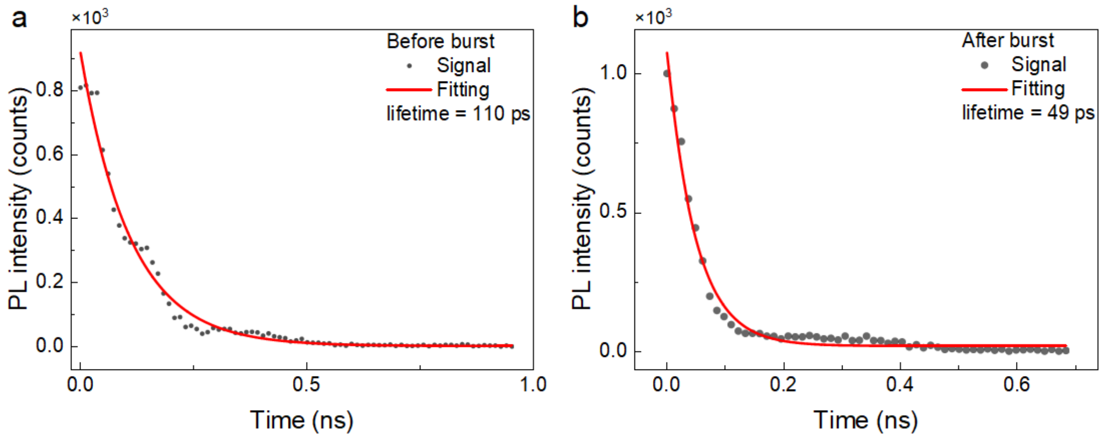

Fig. S24 Luminescence decays measured for a Si/SiO_2 cuboid (l = 600 nm, w = 340 nm) at λ = 600 nm before (a) and after (b) the luminescence burst. In each case, the luminescence lifetime is extracted by fitting the luminescence decay with an exponential function.

Note 15 Size dependence of luminescence intensity

In Fig. S25a, we present the scattering spectra measured for Si/SiO_2 cuboids with different lengths ranging from l = 600 nm to l = 640 nm. The evolution of the F-P mode with increasing length of the Si/SiO_2 cuboid is indicated by the dashed line. We used femtosecond laser pulses to excite the Si/SiO_2 cuboids. The excitation wavelength, which was chosen to be 720 nm, is marked by the solid line. The luminescence intensities observed for Si/SiO_2 cuboids at different excitation pulse energies are presented in Fig. S25b. It is noticed that the highest excitation efficiency is achieved when the quasi-BIC matches the excitation wavelength.

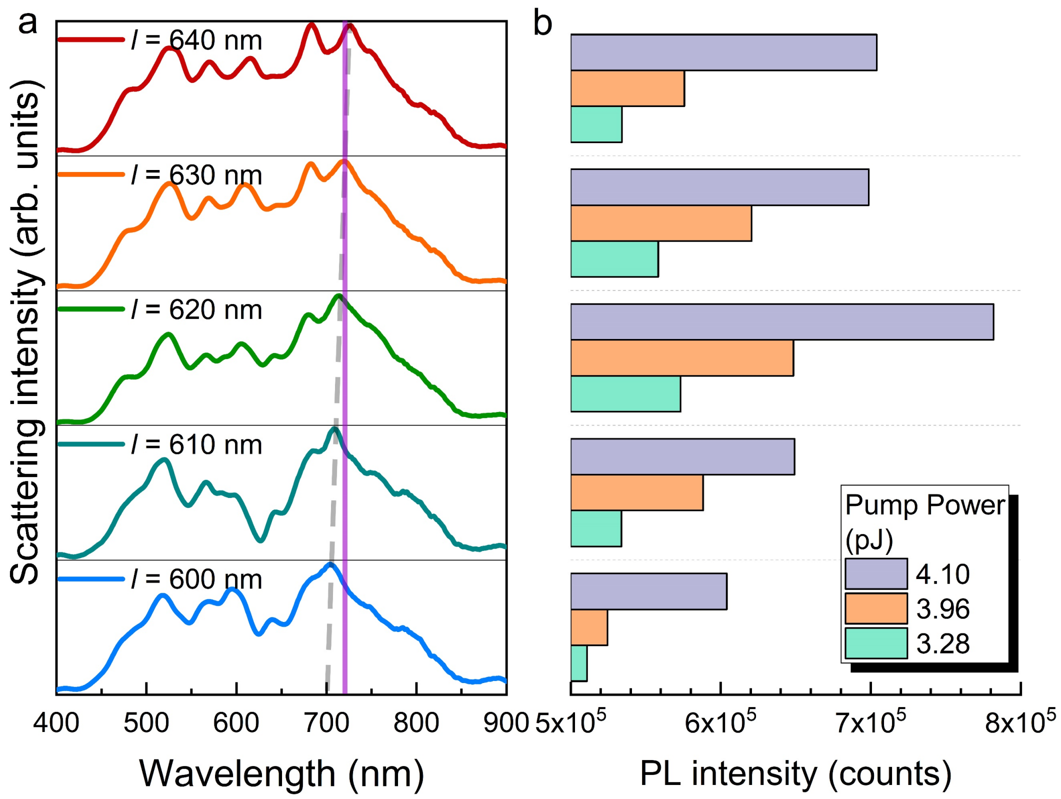

Fig. S25 (a) Scattering spectra measured for Si/SiO_2 cuboids with different lengths (w = 340 nm, h = 230 nm). (b) Integrated luminescence intensities observed for Si/SiO_2 cuboids at different pump powers.

Note 16 Luminescence from arrays of Si/SiO_2 cuboids

In Fig. S26, we show the luminescence images of the Si/SiO_2 cuboid arrays obtained by using two-photon laser scanning confocal microscope with different polarizations (TE and TM) and at different pulse energies of 2.05, 4.1, and 6.15 pJ. The excitation wavelength was chosen to be 720 nm. It can be seen that excitation efficiency for the TE-polarized light is much larger than that for the TM-polarized light. When TE-polarized light was used, about one-third of the Si/SiO_2 cuboids in the scanning region were lightened up at low pulse energy of 2.05 pJ. In contrast, the Si/SiO_2 cuboid arrays were invisible when TM-polarized light was employed. At high pulse energy of 6.15 pJ, bright luminescence was observed from most Si/SiO_2 cuboids for the TE-polarized light. In comparison, only a small part of the Si/SiO_2 cuboids become visible for the TM-polarized light.

In addition, it is noticed that no luminescence from the markers was observed in all cases, implying the importance of the optical resonances in the enhancement of the luminescence.

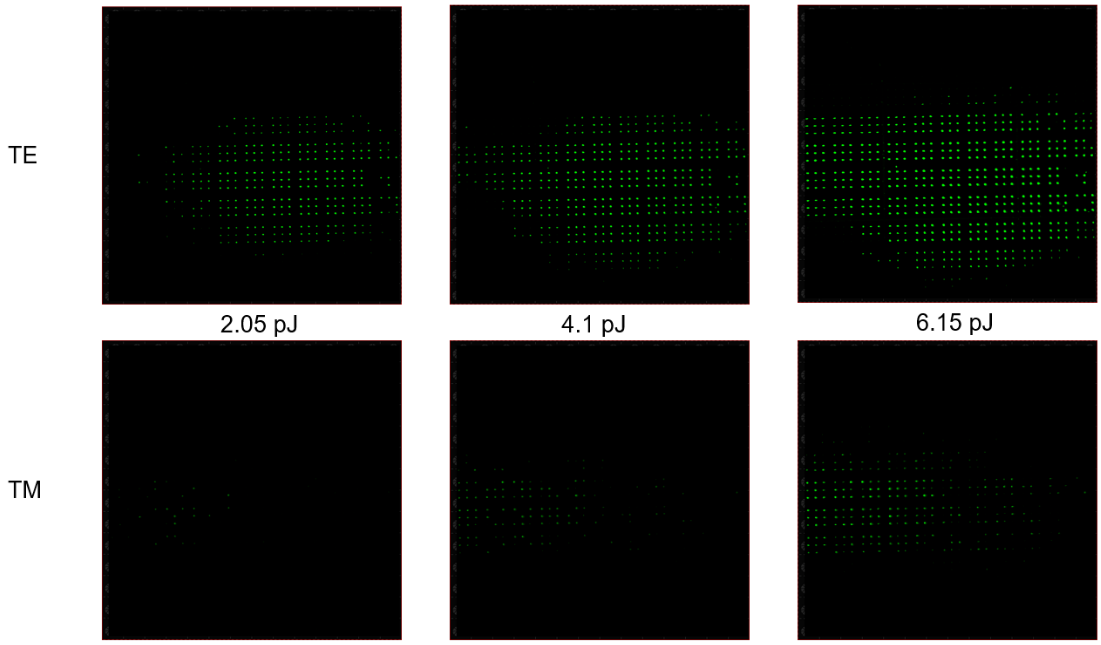

Fig. S26 Luminescence images of the Si/SiO_2 cuboid arrays obtained by using two-photon confocal laser scanning microscope at a wavelength of 720 nm. The pulse energy and laser polarization used in the scanning are indicated in the images.

Note 17 Optical characterization

The linear and nonlinear optical responses of Si cuboids with different geometrical parameters are characterized in this work.

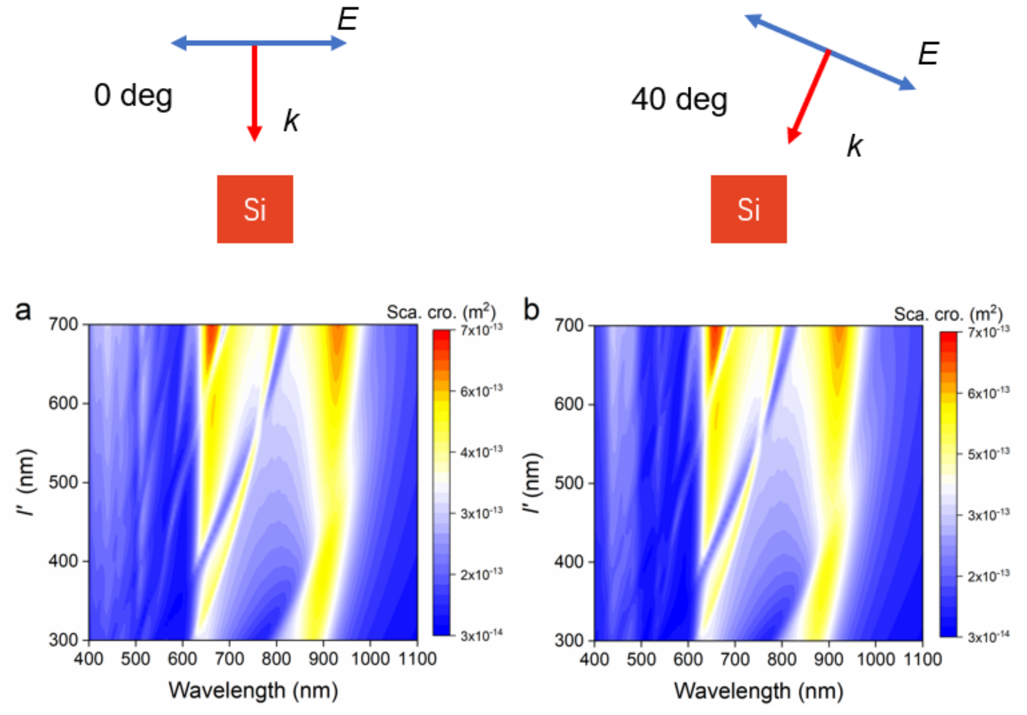

Fig. S27 Scattering spectra calculated for Si cuboids with different lengths, which are illuminated by using plane waves at normal (a) and oblique (b) incidences.

The scattering spectra of Si cuboids were measured by using a dark-field microscope with a home-built oblique incidence system. In this case, the illumination light is incident on Si cuboid at an angle of ~40° and the forward scattering light was collected by the objective of the dark-field microscope. In Fig. S27, we compare the scattering spectra calculated for Si cuboids with different lengths illuminated by using plane waves at normal (0°) and oblique (40°) incidences. One can see that the influence of the incidence angle on the optical modes and their evolutions are quite small, especially in the long wavelength range ($\lambda > 500$ nm).

References

1 Aspnes, D. E. & Studna, A. A. Dielectric functions and optical parameters of Si, Ge, GaP, GaAs, GaSb, InP, InAs, and InSb from 1.5 to 6.0 eV. *Phys. Rev. B* **27**, 985–1009(1983).

2 Limonov, M. F., Rybin, M. V., Poddubny, A. N. & Kivshar, Y. S. Fano resonances in photonics. *Nat. Photonics* **11**, 543-554(2017).

3 Mylnikov, V. *et al.* Lasing Action in Single Subwavelength Particles Supporting Supercavity Modes. *ACS Nano* **14**, 7338–7346(2020).

4 Cullis, A. G., Canham, L. T. & Calcott, P. D. J. The structural and luminescence properties of porous silicon. *Journal of Applied Physics* **82**, 909–965(1997).

5 Credo, G. M., Mason, M. D. & Buratto, S. K. External quantum efficiency of single porous silicon nanoparticles. *Applied Physics Letters* **74**, 1978–1980(1999).

6 Park, N.-M., Kim, T.-S. & Park, S.-J. Band gap engineering of amorphous silicon quantum dots for light-emitting diodes. *Applied Physics Letters* **78**, 2575–2577(2001).

7 Anthony, R. & Kortshagen, U. Photoluminescence quantum yields of amorphous and crystalline silicon nanoparticles. *Physical Review B* **80**, 115407(2009).

8 Dohnalová, K. *et al.* Surface brightens up Si quantum dots: direct bandgap-like size-tunable emission. *Light: Science & Applications* **2**, e47–e47(2013).

9 Dohnalova, K. *et al.* Microscopic origin of the fast blue-green luminescence of chemically synthesized non-oxidized silicon quantum dots. *Small* **8**, 3185–3191(2012).

10 Valenta, J., Greben, M., Gutsch, S., Hiller, D. & Zacharias, M. Effects of inter-nanocrystal distance on luminescence quantum yield in ensembles of Si nanocrystals. *Applied Physics Letters* **105**, 243107(2014).

11 Miller, J. B. *et al.* Ensemble Brightening and Enhanced Quantum Yield in Size-Purified Silicon Nanocrystals. *ACS Nano* **6**, 7389–7396(2012).

12 Jurbergs, D., Rogojina, E., Mangolini, L. & Kortshagen, U. Silicon nanocrystals with ensemble quantum yields exceeding 60%. *Applied Physics Letters* **88**, 233116(2006).

13 Mangolini, L., Jurbergs, D., Rogojina, E. & Kortshagen, U. Plasma synthesis and liquid-phase surface passivation of brightly luminescent Si nanocrystals. *Journal of Luminescence* **121**, 327–334(2006).

14 Limpens, R. & Gregorkiewicz, T. Spectroscopic investigations of dark Si nanocrystals in SiO2 and their role in external quantum efficiency quenching. *Journal of Applied Physics* **114**, 074304(2013).

15 Sangghaleh, F., Sychugov, I., Yang, Z., Veinot, J. G. C. & Linnros, J. Near-Unity Internal Quantum Efficiency of Luminescent Silicon Nanocrystals with Ligand Passivation. *ACS Nano* **9**, 7097–7104(2015).

16 Trinh, M. T., Limpens, R. & Gregorkiewicz, T. Experimental Investigations and Modeling of Auger Recombination in Silicon Nanocrystals. *The Journal of Physical Chemistry C* **117**, 5963–5968(2013).

17 Mastronardi, M. L. *et al.* Size-dependent absolute quantum yields for size-separated colloidally-stable silicon nanocrystals. *Nano Lett* **12**, 337−342(2012).
18 Timmerman, D., Valenta, J., Dohnalova, K., de Boer, W. D. & Gregorkiewicz, T. Step-like enhancement of luminescence quantum yield of silicon nanocrystals. *Nat Nanotechnol* **6**, 710–713(2011).
19 Brown, S. L. *et al.* Abrupt Size Partitioning of Multimodal Photoluminescence Relaxation in Monodisperse Silicon Nanocrystals. *ACS Nano* **11**, 1597–1603(2017).
20 Walters, R. J., Kalkman, J., Polman, A., Atwater, H. A. & de Dood, M. J. A. Photoluminescence quantum efficiency of dense silicon nanocrystal ensembles in SiO2. *Physical Review B* **73**, 132302(2006).
21 Miura, S., Nakamura, T., Fujii, M., Inui, M. & Hayashi, S. Size dependence of photoluminescence quantum efficiency of Si nanocrystals. *Physical Review B* **73**, 245333(2006).
22 Kalkman, J., Gersen, H., Kuipers, L. & Polman, A. Excitation of surface plasmons at a SiO2 aginter face by silicon quantum dots: Experiment and theory. *Physical Review B* **73**, 075317(2006).
23 Sankaran, R. M., Holunga, D., Flagan, R. C. & Giapis, K. P. Synthesis of Blue Luminescent Si Nanoparticles Using Atmospheric-Pressure Microdischarges. *Nano Lett* **5**, 537–541(2005).
