# Supplementary material for: Highly efficient nonlinear optical emission from a subwavelength crystalline silicon cuboid mediated by supercavity mode
Source: Nat Commun. 2022 May 18;13:2749. doi: 10.1038/s41467-022-30503-4 (PMC9117321; doi:10.1038/s41467-022-30503-4)
Supplement: Supplementary file 3 — Description to Additional Supplementary Information [file 41467_2022_30503_MOESM3_ESM.pdf]

Description of Additional Supplementary Files

Move 1: Evolution of the hot electron luminescence from a Si cuboid with increasing excitation pulse energy recorded by using a CCD. It shows clearly the luminescence burst when the excitation pulse energy exceeds a critical value (i.e., the threshold).

Move 2: Evolution of the hot electron luminescence from another Si cuboid with increasing excitation pulse energy recorded by using a CCD. It shows clearly the luminescence burst when the excitation pulse energy exceeds a critical value (i.e., the threshold).
